# Daily Activity Patterns and Co-Occurrence of Duikers Revealed by an Intensive Camera Trap Survey across Central African Rainforests

**DOI:** 10.3390/ani10122200

**Published:** 2020-11-24

**Authors:** Fructueux G. A. Houngbégnon, Daniel Cornelis, Cédric Vermeulen, Bonaventure Sonké, Stephan Ntie, Adeline Fayolle, Davy Fonteyn, Simon Lhoest, Quentin Evrard, Fabrice Yapi, François Sandrin, Liliana Vanegas, Idriss Ayaya, Clément Hardy, Sebastien Le Bel, Jean-Louis Doucet

**Affiliations:** 1 Terra Teaching and Research Centre, Forest Is life, Gembloux Agro-Bio Tech, Université de Liège, Passage des Déportés 2, BE-5030 Gembloux, Belgium; cvermeulen@uliege.be (C.V.); adeline.fayolle@uliege.be (A.F.); davy.fonteyn@doct.uliege.be (D.F.); simlho@hotmail.com (S.L.); q.evrard@doct.ulg.ac.be (Q.E.); jldoucet@uliege.be (J.-L.D.); 2 French Agricultural Research Center for International Development (CIRAD), 34398 Montpellier CEDEX 5, France; daniel.cornelis@cirad.fr (D.C.); sebastien.le_bel@cirad.fr (S.L.B.); 3 Département de Biologie, Laboratoire de Botanique Systématique et d’Écologie, École Normale Supérieure, Université de Yaoundé I, BP 047 Yaoundé, Cameroon; bonaventuresonke@ens.cm; 4 Département de Biologie, Laboratoire de Biologie Moléculaire et Cellulaire (LABMC), Université des Sciences et Techniques de Masuku (USTM), BP 941 Franceville, Gabon; stephanntie@yahoo.fr; 5 Office Ivoirien des Parcs et Réserves, Yamoussoukro 225, Cote D’Ivoire; fabbyof@yahoo.fr; 6 Independent Consultants, Center for International Forestry Research (CIFOR), Situ Gede, Bogor Barat, Bogor Jawa 16115, Barat, Indonesia; f.sandrin1990@gmail.com (F.S.); lilovan7@gmail.com (L.V.); 7 Faculté des Sciences Agronomiques, Université de l’Uélé, Haut-Uélé, Isiro BP 670, Congo; idrissayaya@gmail.com; 8 Department of Biological Sciences, UQAM, Montréal, QC H3W 1R7, Canada; clem.hardy@outlook.fr

**Keywords:** daily activity patterns, duikers, Central Africa, overlap coefficient, co-occurrence, camera traps

## Abstract

**Simple Summary:**

Forest duikers are one of the most important groups of mammals in Central African rainforests and over the whole Guineo-Congolian Region. To better understand the mechanisms of habitat use and sharing among duiker species, we quantified duiker activity, temporal and spatial interactions. Data were collected using camera traps over five years, in 12 sites scattered in four countries: Cameroon, Congo, Democratic Republic of Congo, and Gabon. We found that duikers usually have two main peaks of activity, which are mostly dependent on sunrise and/or sunset. In addition, analyses of temporal interactions (for five species) identified four species pairs with strong interactions and six pairs with weak interactions. Spatial interaction tests revealed no competitive habitat use among species. Our results contribute to a better understanding of the ecology of duikers in Central African rainforests, in order to improve their conservation and management.

**Abstract:**

The duiker community in Central African rainforests includes a diversity of species that can coexist in the same area. The study of their activity patterns is needed to better understand habitat use or association between the species. Using camera traps, we studied the temporal activity patterns, and quantified for the first time the temporal overlap and spatial co-occurrence between species. Our results show that: (i) Two species are strongly diurnal: *Cephalophus leucogaster*, and *Philantomba congica,* (ii) two species are mostly diurnal: *C.* *callipygus* and *C. nigrifrons,* (iii) one species is strongly nocturnal: *C.* *castaneus,* (iv) and one species is mostly nocturnal: *C.* *silvicultor*. Analyses of temporal activities (for five species) identified four species pairs that highly overlapped ($\hat{\Delta }$ ≥ 0.80), and six pairs that weakly overlapped ($\hat{\Delta }$ between 0.06 and 0.35). Finally, co-occurrence tests reveal a truly random co-occurrence (*p_lt_* > 0.05 and *p_gt_* > 0.05) for six species pairs, and a positive co-occurrence (*p_gt_* < 0.05) for four pairs. Positive co-occurrences are particularly noted for pairs formed by *C.* *callipygus* with the other species (except *C. nigrifrons*). These results are essential for a better understanding of the coexistence of duikers and the ecology of poorly known species (*C. leucogaster* and *C. nigrifrons*), and provide clarification on the activity patterns of *C. silvicultor* which was subject to controversy. Camera traps proved then to be a powerful tool for studying the activity patterns of free-ranging duiker populations.

## 1. Introduction

Duikers are endemic antelope species to sub-Saharan Africa. According to the herbivome concept (delimitation of different large mammal herbivory/frugivory regimes, analogous to biome concept) of Hempson et al. [1], the herbivome of duikers covers forested tropical regions. The number of species of duikers is still controversial because of their unresolved phylogenetic linkages [2,3,4]. In addition, there is controversy over the taxonomy of duikers. Indeed, taxa formerly classified as subspecies have recently been elevated to the rank of full species by Groves and Grubb [5], (Table S1). Three genera of duikers are currently recognized (*Cephalophus*, *Philantomba*, and the monotypic savanna specialist *Sylvicapra*) [4]. Overall, duikers can be organized into three groups according to their coat colour and their size: (1) Blue duikers (small duikers), (2) red duikers (medium duikers), (3) and yellow-backed duiker (large duiker) [6,7] (Table S1). Some taxa within these groups (such as *C. crusalbum* and *C. callipygus*) are suggested to be close phylogenetically [3,8]. According to Ntie et al. [3], *C. callipygus* and *C. crusalbum* are not monophyletic. This lack of monophyly may be due to incomplete lineage sorting commonly observed in recently derived taxa, hybridization or the presence of nuclear translocated copies of mitochondrial DNA. In fact, *C. crusalbum* was distinguished from *C. callipygus* with striking white lower legs [9]. However, Hedwig et al. [10] observed phenotypic variations that make them indistinguishable. The duiker community is the most heavily hunted community in the Congo Basin [11,12]. They represent a very significant part of the animal biomass in forest ecosystems [1,13], and up to seven species of duikers can live sympatrically [14].

Daily activity patterns inform the movement ecology that regulates physiology of individuals and growth of a population, and is an important attribute of species coexistence [15,16]. The study of activity patterns provides insight into the ecological processes that shape the use of space by an animal community, including: Shared home range, food resource use (competitive or segregated), predation and energy expenditure [17,18]. In general, competition between sympatric species is mitigated by partitioning resources in three niche dimensions: Space, time and food [19]. At larger scales, cohabitation between species is facilitated through spatial segregation [20]. Thus, geographic range selection (*first-order selection*), and home range (*second-order*) are the primary factors that reduce competition [21]. At finer scales, co-occurrence is facilited by selection of feeding site (*third-order*) within the home range and finally the selection of food items (*fourth-order*) [21]. Moreover, time is appropriately partitioned between species in order to avoid agonistic or competitive encounters [22]. The daily activity patterns of terrestrial mammals can be categorized into nocturnal, diurnal, crepuscular, and cathemeral (active during hours of daylight and darkness) [23]. Ridout and Linkie [24] developed a statistical method (initially proposed by Weitzman [25]) for the study of temporal interactions between species, estimating the overlap coefficient between two activity patterns. Probabilistic models for analysing of species spatial co-occurrence have also been developed [26,27].

Changes in activity patterns, particularly as a result of anthropogenic activities, can result in physiological stress that can affect reproduction and survival of individual animals, and therefore population growth [28]. Understanding activity patterns is thus fundamental to establishing effective strategies for the conservation and sustainable management of animal communities [29]. Most of the studies concerning the activity patterns of mammals have been conducted on animals held in captivity using direct observations [30,31]. Indeed, studying the activity patterns of animal populations in their natural environment is often difficult, and even more so in rainforests [32]. Activity patterns in the natural environment can be assessed using activity loggers, GPS or VHF collars, but this invasive method requires capturing and equipping animals [32,33]. Less invasive and more affordable, camera traps are now massively used in tropical forests and provide large datasets on the activity of a wide range of species [29,34]. Camera traps generate time-stamped information from each photo accumulated over time, which helps to identify activity periods of different species [17,18,35]. However, the biological characteristics of the species (weight, age, sex) are difficult to observe.

The daily activity patterns of duikers in the moist forest of Central Africa have been studied on only a few individual species, by telemetry (*P. congica*, *C. callipygus*, and *C. castaneus*) [33,36,37], by observations in captivity (*C. silvicultor*) [38,39], and summarily by camera trapping (*P. congica*, *C. callipygus*, *C. castaneus*, *C. leucogaster*, *C. crusalbum*, *C. silvicultor*, and *C. nigrifrons*) [10,40,41]. To date, the activity patterns of several species remains unquantified.

In this paper, we first characterize the activity patterns of the duiker community in the rainforests of Central Africa through the use of camera traps. We then investigate the interspecific relationships, studying temporal overlap of activities (temporal interactions) and co-occurrence (spatial interactions). Since duikers vary greatly in size, greatly constraining fruit selection [14,42], we expected a higher spatio-temporal segregation between species of similar size (red duikers).

Understanding activity patterns and niche partitioning may help scientists and managers to elucidate ecological mechanisms which allow the co-existence of duikers, thereby benefitting conservation and management of duiker community.

## 2. Materials and Methods

### 2.1. Study Area

**Figure 1:**
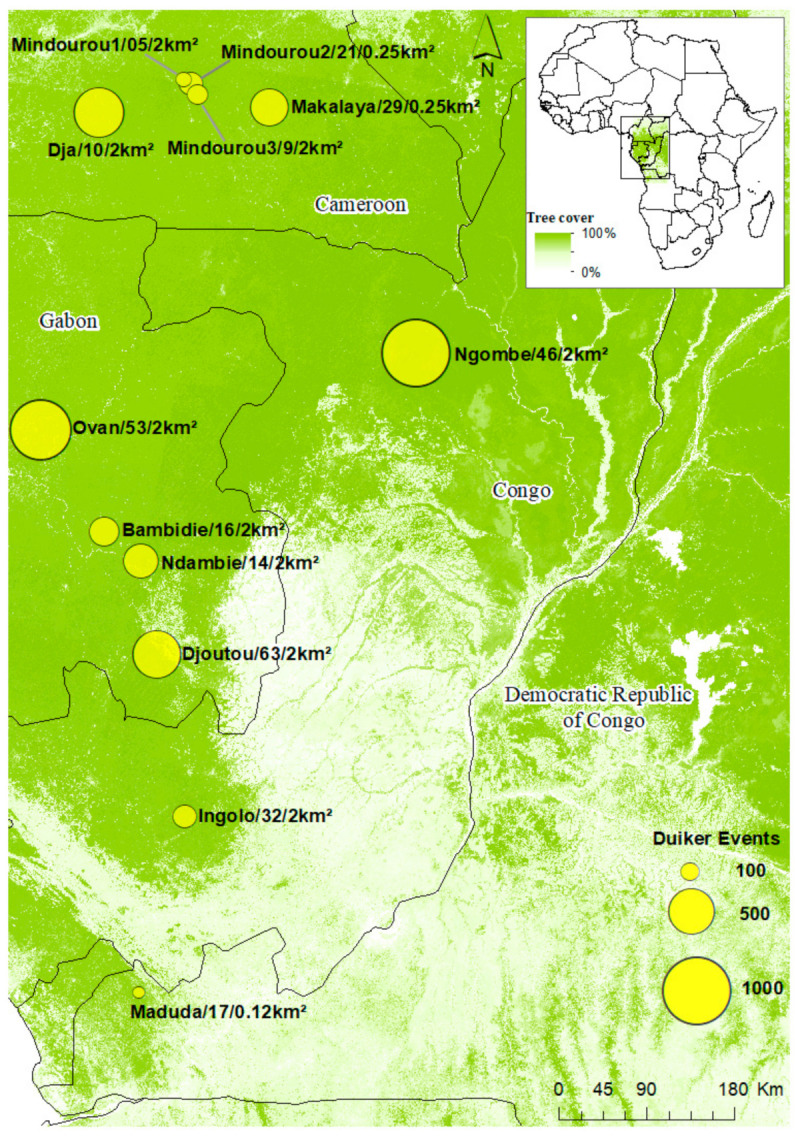
Location of the study sites. Legend: Name of site/Number of cameras/Camera density. Tree cover 2000 [52].

The study area covers the rainforests of Central Africa between 5° S–4° N and 12° E–16° E. It includes 12 sites located in four countries: Cameroon (five sites), Gabon (four sites), Congo (two sites), and the Democratic Republic of Congo (one site) (Figure 1). The camera traps used to determine the activity patterns of duikers were set up along a north-south gradient encountering most vegetation types representative of the Guinea-Congolese forests [43]. The climate is equatorial, with average rainfall ranging from 1400 mm to 1700 mm/year [44].

### 2.2. Camera Traps Data Collection

Overall, 428 camera traps were installed at the 12 sites over a five-year period (2014, 2016–2019, see Table S2 for details). Different models of camera traps (Bushnell Trophy Cam HD and Moultrie Game Spy) with passive infrared sensors were used.

Camera trap locations were predetermined using systematic sampling. They were deployed at a density of one camera per 0.12 km^2^ to 2 km^2^ according to the size of the sites (see Figure 1). Specific sites for camera placement were selected using predefined GPS coordinates. Cameras were installed at the base of a tree at an average height of 30 cm above the ground, and were oriented towards animal tracks or open spaces, without using bait [45,46]. In order to reduce false triggers, and allow a good identification of species, the cover of grasses and lianas were slightly reduced in the camera’s field of vision within a radius of 3 m. The cameras were set to the local times of each sampling site (UTC+1) and set to take one to three photos per shutter released at intervals of one to three seconds. They were activated in photo or hybrid (photo + video) mode and operated 24 hours a day for one to four months generating a total effective sampling effort of 17,827 trap nights.

For this study, we only selected camera traps that captured at least one clearly identifiable species of duiker. As a result, only 315 camera traps (73.6%) were retained for analysis purposes. The resulting photos were processed in Camera base [47]. The following criteria were met for the selection of independent events:

- The species was clearly identified. The identification was made on the basis of the physical traits described by Groves et al. [48] and Castello [9]. In the absence of consensus we used the taxonomy proposed by Groves and Grubb [5] (Table S1).
- A photo of several individuals (multiple individuals in the same image) of the same species was treated as a single individual event [49].
- To avoid pseudo-replication, an interval of one hour was considered in order to identify independent events in the same species at the same camera-trap location [50,51].
- A photo of two species was treated as two separate independent events.

### 2.3. Statistical Analysis

#### 2.3.1. Activity Periods

Any individual caught on a camera trap was considered to be active at that time [18]. The sunrise and sunset times for each site were obtained from the website of the Department of Astronomical Applications of the United States Naval Observatory [53]. For all sites, the average sunrise and sunset were 6:00 h and 18:00 hrespectively (Table S2). An individual activity was therefore classified as diurnal if photographed between sunrise and sunset (6:00 h–17:59 h), and as nocturnal if photographed between sunset and sunrise (18:00 h–5:59 h) [15,17].

Following the classification used by Gómez et al. [54] and Azevedo et al. [15], we have defined a taxon as: (1) Strongly diurnal or nocturnal if at least 90% of observations were made during the day or the night respectively, (2) mostly diurnal if between 10 and 29% of the observations were during the night, (3) mostly nocturnal if between 70 and 89% of the observations were obtained during the night, and (4) cathemeral if between 30 and 69% of the observations were recorded during the night. In addition, in order to characterise twilight activities, we considered the twilight period to be the time interval between 1 hour before and 1 hour after sunrise and sunset, respectively [55].

The gross distribution of the observations of each species was specified by a pie chart. In this type of graph, each observation is represented by a dot around the circumference of the hourly circle. A rose chart was also reproduced to show the relative frequency of observations for each time slot. The median time of activity was estimated and represented by a vector of identical length for each species [56]. The new Hermans-Rasson test (1000 bootstrap replicates) was carried out in order to check whether the observations of each species were evenly distributed around the time circle (uniform distribution) [57]. The activity profile of each species was modelled by estimating kernel density and non-negative trigonometric sums [58]. Lashley et al. [16] recommended a sample size ~100 detections or more for activity patterns study. However, we used kernel density and non-negative trigonometric sums to assess sample size, following recommendations of Linkie and Ridout [59]. Therefore, if for any given species there is a large difference between the trends of kernel density and non-negative trigonometric sums, this implies that the sample size for that species is too small to reliably explain the activity [59]. Finally, we estimated the activity level of duikers (the proportion of time that duikers spend active) using the method developed by Rowcliffe et al. [18].

#### 2.3.2. Overlap of Activity Patterns

The quantification of the overlap in activity patterns between two sympatric species was done using the overlap coefficient ($\hat{\Delta }$) [24]. The coefficient $\hat{\Delta }$ can be defined as the area under the curve that is formed by taking the minimum of two density functions (Kernel density) at each point in time [17]. It varies from 0 (no overlapping activity patterns) to 1 (identical activity patterns). We considered that two sympatric species had a strong overlap in activity patterns if $\hat{\Delta }$ > 0.75, moderate if 0.5 > $\hat{\Delta }$ ≥ 0.75, and low if $\hat{\Delta }$ ≤ 0.5 [60].

Based on the recommendations of Meredith and Ridout [61], $\hat{\Delta }$_1_ was used if of the two samples compared, the smaller of the two samples was less than 75 observations, otherwise $\hat{\Delta }$_4_ was applied. For two probability density function *f*(.) and *g*(.), $\hat{\Delta }$_1_ and $\hat{\Delta }$_4_ can be written in the following form [24]:

(1) $${\hat{\Delta }}_{1}=\int_{0}^{1}\min\;\left\{\hat{f}\left(t\right),\;\hat{g}\left(t\right)\right\}\mathrm{dt}$$

(2) $${\hat{\Delta }}_{4}=\frac{1}{2}\left(\frac{1}{n}\overset{n}{\underset{i=1}{\sum }}\min\left\{1,\frac{\hat{g}\left(x_{i}\right)}{\hat{f}\left(x_{i}\right)}\right\}+\frac{1}{m}\overset{m}{\underset{i=1}{\sum }}\min\left\{1,\frac{\hat{f}\left(y_{i}\right)}{\hat{g}\left(y_{i}\right)}\right\}\right).$$

where *n*: Total number of observations of the first species, *m*: Total number of observations of the second species; *x_1_*,...,*x_*n*_* and *y_1_*,...,*y_*m*_* represent the time series of the two samples.

We performed a Bootstrap test (1000 iteractions) to complement the overlap coefficient information, and see whether two sets of circular observations were from the same distribution [62]. The confidence intervals (for activity level, and coefficient $\hat{\Delta }$) were calculated as percentile intervals from 1000 bootstrap samples [61].

#### 2.3.3. Spatial Co-Occurrence Patterns

In order to assess the spatial interactions between the different species of duikers, statistical co-occurrence tests were carried out. They were based on two probabilities: (1) The probability (*p_lt_*) that two species co-occur at a frequency lower than the observed co-occurrence frequency, (2) and the probability (*p_gt_*) that two species co-occur at a frequency higher than the observed co-occurrence frequency [26]. If *p_lt_* < 0.05 or *p_gt_* < 0.05, this implies negative co-occurrence (competitive interaction) and positive co-occurrence (no antagonism) respectively for the considered species pairs. However, when *p_lt_* > 0.05 and *p_gt_ > * 0.05, co-occurrence is said to be truly random (independent distribution) [26,46,63]. *p_lt_* and *p_gt_* probabilities are defined as follows [26]:

(3) $$P_{lt}=\sum P_{j}\;\mathrm{for}\;j=0\;\mathrm{to}\;Q_{obs}-1$$

(4) $$P_{gt}=\sum \;P_{j}\;\mathrm{for}\;j=\;Q_{obs}+1\;\mathrm{to}\;N$$

with *Q_obs_* the frequency of co-occurrence observed, *N* the total number of camera stations, and *P_j_* the probability that two species co-occur at exactly *j* camera stations (*j* = 0 to *N*). *P_j_* is obtained by the following equation [63]:

(5) $$P_{j}=\frac{\left(\begin{array}{c} N_{1}\\ j \end{array}\right)\times \left(\begin{array}{c} N-N_{1}\\ N_{2}-j \end{array}\right)}{\left(\begin{array}{c} N\\ N_{2} \end{array}\right)}$$

where *N*_1_ is the number of camera stations where species 1 occurs, and *N*_2_ the number of camera stations where species 2 occurs. The term $\left(\begin{array}{c} N_{1}\\ j \end{array}\right)$ represents the number of ways of selecting *j* camera stations that have species 1 given that there are *N*_1_ such camera stations in the “population” of all camera stations. The term $\left(\begin{array}{c} N-N_{1}\\ N_{2}-j \end{array}\right)$ represents the number of ways of selecting $N_{2}-j$ camera stations that have species 2 but not species 1, given that there are $N-N_{1}$ such camera stations. Multiplying the numerator together gives the total number of ways of selecting *j* camera stations that have species 1 and 2. The term $\left(\begin{array}{c} N\\ N_{2} \end{array}\right)$ represents the total number of ways *N*_2_ camera stations could be obtained out of a total of *N* camera stations. Thus, the equation is giving the proportion of *N*_2_ camera stations that also have species 1 under the condition that the two species co-occur at *j* camera stations [63].

The standardised effect sizes were also calculated. They were obtained by the difference between the observed and expected co-occurrence frequencies divided by the number of sampling points (315 camera stations) [26]. Varying from −1 to 1, it has the advantage of facilitating the comparison of results between different studies or methods [26,46].

Finally, we performed the Mantel test to evaluate the correlation between spatial co-occurrence and temporal overlap patterns.

All statistical analyses were performed using R software [64] with the packages “ade4” [65], “CircMLE [66]”, “cooccur” [63], “circular” [67], “overlap” [61], and “activity” [68].

## 3. Results

### 3.1. Inventory Data

A total of 4358 independent detection events (32% of duiker photos analyzed) were recorded and corresponded to six species of duiker. The most detected species was *P. congica* (*n* = 2562), while the least was *C. nigrifrons* (*n* = 42) (Table 1).

Because of their close phylogenetic relationship, *C. crusalbum* and *C. callipygus* are considered to be the same species (under *C. callipygus*, *n* = 945). We did not take *C. nigrifrons* into account in the analyses of temporal overlap and spatial co-occurrence, since it is known that its habitat is completely different from other species (swampy area) [9,69]. All six species are sympatric in the Makalaya, Bambidie, Djoutou, and Ovan sites.

### 3.2. Species-Specific Activity Periods

**Figure 2:**
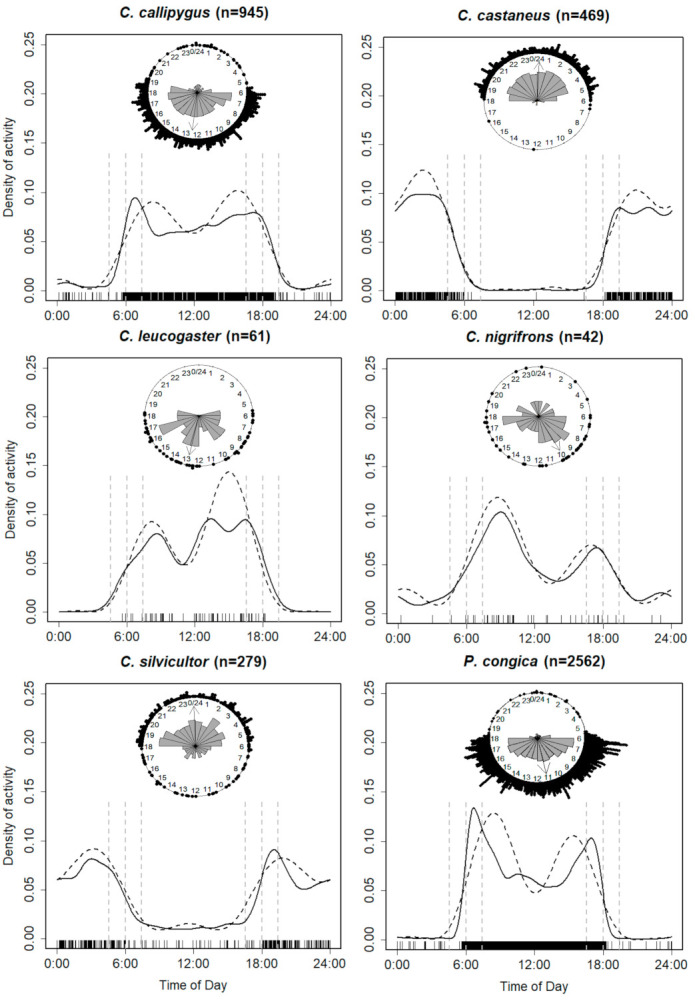
Density estimates of daily activity patterns of six duiker species from the Central African rainforests. Solid lines indicate the kernel-density estimates; Dashed lines indicate the trigonometric sum distributions; Short vertical lines above the x-axis indicate the times of individual photographs. The six dotted vertical lines indicate, start of twilight, sunrise, end of twilight, start of twilight, sunset, end of twilight, respectively. Raw circular plot of activity patterns of each duikers is represented inset. Sectors of the rose diagram indicate relative frequencies in the 24 class intervals; arrows indicate the median: 12.5, 24.2, 12.9, 10.1, 23.9, and 11.1 hours, respectively.

The new Hermans-Rasson test indicates a non-uniform distribution of observations for each taxon (*p* < 0.01). Trends in kernel density and trigonometric sum showed little differences for each species. The species thus present sufficient observations for the study of activity patterns. All species show bimodal activity (Figure 2).

Species with strong diurnal activity were: *P. congica* (97%, *n* = 2474), and *C. leucogaster* (90%, *n* = 55). Their twilight activities accounted for a proportion of 26% (*n* = 666), and 21% (*n* = 13), respectively. The mostly diurnal species *C. callipygus* (86%, *n* = 816) and *C. nigrifrons* (79%, *n* = 33) have a twilight activity of 30% (*n* = 278) and 24% (*n* = 10) respectively. Two nocturnal species were observed: *C. castaneus* (strongly nocturnal, 99%, *n* = 465) with a twilight activity of 11% (*n* = 51), and *C. silvicultor* (mostly nocturnal, 85%, *n* = 236) with a twilight activity of 21% (*n* = 59).

The median activity of diurnal species is found between 10:00 and 13:00, while that of nocturnals was at midnight (Figure 2). The least activity level was recorded for *P. congica* (0.31 CI: 0.31–0.37) and the largest for *C. silvicultor* (0.46 CI: 0.41–0.58). The activity levels of the other species were: 0.40 (CI: 0.25–0.57) *C. nigrifrons*, 0.42 (CI: 0.36–0.45) *C. castaneus*, 0.44 (CI: 0.28–0.46) *C. leucogaster*, and 0.44 (CI: 0.44–0.54) *C. callipygus* (Figure S1).

Furthermore, duikers were rarely observed in groups. For instance, *C. leucogaster* was always photographed alone. Simultaneous observations of two individuals accounted for only 1% of the observations for *C. castaneus*, 2% for *C. callipygus* and *C. silvicultor*, and 4% for *C. nigrifrons*. However, for *P. congica*, this percentage is much higher, reaching 13%. Moreover, *P. congica* was the only species observed with three individuals (1%). In addition, a mixed species group consisting of *C. callipygus* and *P. congica* was observed on three occasions.

### 3.3. Temporal Overlap Patterns

**Figure 3:**
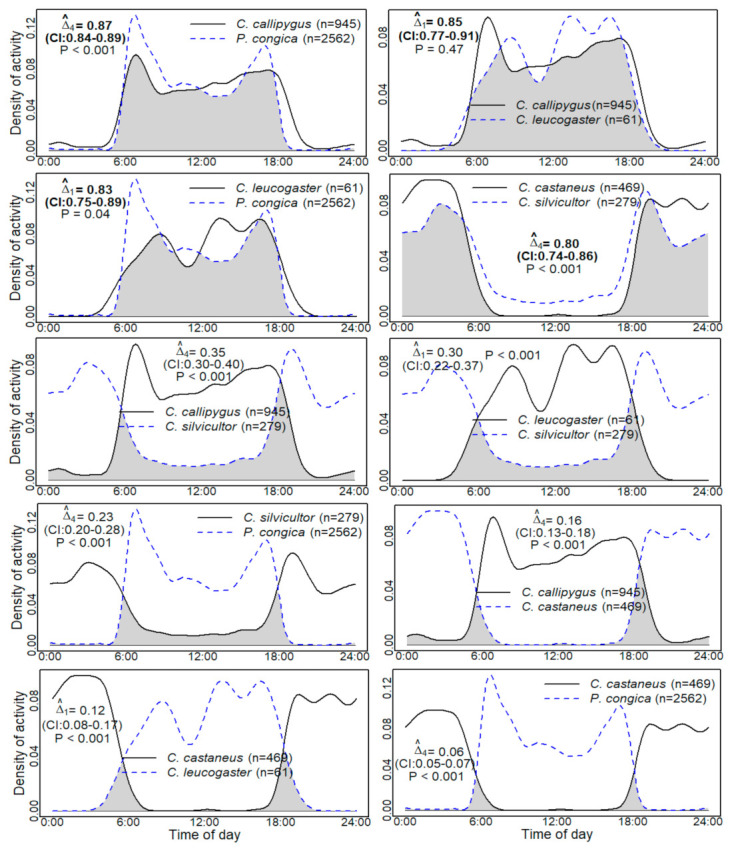
Temporal overlap of duikers of the Central African rainforest. The overlap coefficient is the shaded area. Approximate 95% bootstrap confidence intervals are in parentheses. High temporal overlaps are in bold type. *p* (*p*-values): Probability that two sets of circular observations come from the same distribution.

Overlap values were computed for all pairs of species and varied from 0.06 (CI: 0.05–0.07) to 0.87 (CI: 0.84–0.89) (Figure 3). Specifically, four pairs of species with strong overlap ($\hat{\Delta }$ ≥ 0.80) were distinguished. These were the two nocturnal species *C. castaneus* and *C. silvicultor*, the diurnal species *C. callipygus* and *P. congica,* and the diurnal species *C. leucogaster* associated respectively with the species *C. callipygus* and *P. congica.* However, the distributions were significantly different for three of them (*p* < 0.05). As was expected, low overlap was observed between diurnal and nocturnal species. Overlap coefficients vary in these cases from 0.06 (*C. castaneus* and *P. congica*) to 0.35 (CI: 0.30–0.40) (*C. callipygus* and *C. silvicultor*). No moderate overlap in activity patterns was obtained.

### 3.4. Spatial Co-Occurrence Patterns

The co-occurrence tests carried out indicated a truly random co-occurrence for six species pairs, and a non-random co-occurrence for four pairs (Table 2). Specifically, four pairs of species showed significant positive co-occurrence. These are the species pairs formed by *C. callipygus* with the species *C. castaneus* (*p_gt_* < 0.001), *C. leucogaster* (*p_gt_* = 0.025), *C. silvicultor* (*p_gt_* < 0.001), and *P. congica* (*p_gt_* = 0.001). All other species associations revealed a random co-occurrence.

The Mantel test indicated no significant correlation between spatial co-occurrence and temporal overlap (r = −0.09, *p* = 0.72).

## 4. Discussion

This study provides information related to the activity patterns of duiker populations through the use of camera traps. It is the first study quantifying duiker activity, temporal overlaps and spatial co-occurrences in Central African rainforests.

In our study, we used the new taxonomy proposed by Groves and Grubb [5], which is based on phylogenetic species concept. However, the taxonomy of bovids is a subject of great controversy. Recent studies seem to raise doubts about the new taxonomy for some bovids [70]. Nevertheless, a consensus among scientists still lacking (see Table S1 for taxonomy used by the International Union for Conservation of Nature “IUCN” for duikers studied). We also considered *C. crusalbum* and *C. callipygus* as the same species [3,8,41]. Analysis of circular distributions of the two taxa did not show any significant differences (results not shown, Watson’s two-sample test, *U^2^* = 0.04, *p* > 0.1, Figure S2).

The activity patterns of the species studied were generally dependent on sunrise and/or sunset, suggesting immediate photoperiodicity [32]. We observed bimodal activity for all species, and distinguished between diurnal (*C. callipygus, C. leucogaster, C. nigrifrons,* and *P. congica*) and nocturnal activity patterns (*C. castaneus* and *C. silvicultor*). Species with bimodal peaks occuring around twilight could be interpreted as crepuscular [46]. The peaks of twilight activity observed in duikers actually implies that their visual apparatus is optimised to function at low light levels (mesopic vision) [71].

Two phases of variable length were observed depending on the species: (1) An active phase interspersed with a decrease in activity (resting period), (2) and an inactive phase which corresponds to the day for a nocturnal species and the night for a diurnal species (Figure 2 and Figure 3). The period of activity can be interpreted as the time taken by an animal for foraging, odour analysis, mate finding, scent marking, defending the territory, and related activities. The resting period, on the other hand, can be equated to the actual rumination or resting time when the animal has stopped moving [36]. Moreover, the fact that *P. congica* and *C. silvicultor* have respectively the smallest and greatest activity level can be explained by their weight. Indeed, large duikers require a longer searching time for fruits to meet their energetic needs [72]. However, Nakashima et al. [41] found a similar proportion of activity for *P. congica* (0.35) and *C. silvicultor* (0.36).

All diurnal species, with the exception of *C. leucogaster* and *C. callipygus,* showed peaks of bigeminus type activity (morning peak greater than evening peak). *Cephalophus leucogaster* showed peaks of the alternating type (larger evening peak), while the trend of peaks differed for *C. callipygus* according to the analytical methods (Figure 2). The decrease in activity in the middle of the day may be due to the increase in temperature as the sun’s rays penetrate the forests [36].

The trends on the activity patterns of *P. congica* (Figure 2) are corroborated by Dubost [33] in Gabon. In the same country, Feer [36] showed that the activity of *C. callipygus* begins at sunrise between 5:00 h and 6:00 h and ends between 18:00 h and 19:00 h. The peaks of activity observed by the author were found between 6:00 h and 10:00 h as well as between 16:00 h and 18:00 h, which is generally consistent with our results.

*Cephalophus leucogaster* and *C. nigrifrons* are among the least-known species [9,48]. Hedwig et al. [10] reported diurnal activity for 11 events of *C. leucogaster* detection. In addition, Gessner et al. [40] indicated daytime use of bays by *C. nigrifrons* (number of detection events not indicated).

The nocturnal species *C. castaneus* shows a more pronounced peak at the end of the night than at the beginning of the night. This observation validates the results of Feer [36], who explains this phenomenon by *C. castaneus’* search for shelter at the end of its activity. The species is said to be selective in its choice of resting habitat.

Until now, there were no concensus on the activity patterns of *C. silvicultor*. Dubost [72] considered that the biophysical characteristics of *C. silvicultor* are quite particular for a strictly nocturnal or strictly diurnal species. This finding was reinforced by Kranz and Lumpkin [38] and Lumpkin and Kranz [39]. Kingdon and Lahm [73] stated that the species is essentially active in the twilight, and may be intermittently active both day and night, while Hedwig et al. [10], and Nakashima et al. [41] identified it as a nocturnal species. Our results showed that *C. silvicultor* is mostly nocturnal (85%, *n* = 236) with one of its main peaks of activity at dusk (crepuscular activity). Whether diurnal or nocturnal, all species showed more or less sporadic activity outside of their usual range of activity (Figure 2 and Figure 3).

Although daylight is the main environmental variable that influences the behaviour and determines the activity profile of mammals [74], other biophysical parameters may come into play and modify to some extent day and night cycles [49]. Our study focused mainly on time-stamped information obtained from the cameras. Climatic variability (temperature, precipitation) has therefore not been taken into account. Indeed, it is conceivable that the activity time of duikers could be extended during periods of lower availability of fruits (dry season), following the search for fruit. Feer [36] reported a slight increase in the home range of *C. callipygus* and *C. castaneus* in the dry season. However, Dubost [33] found no increase in the home range of *P. congica* in any season. The later author noted, based on a study carried out on a single *P. congica* individual, bimodal patterns of the bigeminus type only in the rainy season.

Furthermore, we did not take into account the level of anthropogenic pressures in the study sites. It is recognised that human activities (especially hunting) affect the behaviour of duiker populations [75,76] and could therefore influence their activity patterns. The activity patterns of duikers could also be conditioned by those of other species. Indeed, several studies have demonstrated some relationships between the activities of predators and their prey [15,35]. Similarly, cases of commensalism could affect activity patterns. For example, *C. crusalbum* (here *C. callipygus*) has been observed eating under trees where monkeys were present, causing fruit to fall [77]. Also, aggressive or gregarious competitors, such as bush pigs may affect activity patterns of duikers. It would therefore be an added value for future studies, to characterize duikers activity patterns, taking into account climatic factors, anthropogenic pressures, predators (leopard, python, chimpanzee, etc.), and the relationships between duikers and the other species of the sites.

Moreover, we noted that *P. congica* has the highest gregarious activity. This can be explained by the fact that this species is the only one that lives in couples [33]. The other species appear to be rather solitary, except during the breeding season [78].

Contrary to our prediction, statistical tests of spatial co-occurrence confirmed, for all species, a non-competitive interaction. Of the ten co-occurrences tested, only four were significantly positive, and involved *C. callipygus* with all the other considered species respectively. Positive co-occurrence very often indicates, for the species in question, a common habitat preference and an absence of strong antagonistic relationships. The three observations of mixed species (*C. callipygus* and *P. congica*) point in this direction. The random co-occurrences indicates that the concerned species are spatially distributed independently from each other [79]. In this study, we used a co-occurrence probabilistic model which does not account for imperfect detection (naïve co-occurrence). Species associations may differ when imperfect detection is considered [27]. However, our sample sizes are large and probably have reduced this bias. For a comparison of results, we suggest for future studies, the use of multispecies occupancy models [27].

Among the three species of the red duikers group (*C. callipygus, C. castaneus,* and *C. leucogaster*), *C. castaneus* adopted a temporal strategy of activity different from the other species, with the lowest overlap of activity: 0.12 (CI: 0.08–0.17, *C. castaneus* and *C. callipygus*), and 0.16 (CI: 0.13–0.18, *C. castaneus* and *C. leucogaster*), confirming our prediction. One might therefore expect a competitive relationship between *C. callipygus* and *C. leucogaster*. However, co-occurrence patterns showed the absence of an antagonistic relationship (positive or random co-occurrence, Table 2). Based on our results, and referring to the framework proposed by Johnson [21], the coexistence of duikers can be explained by different selection scales: (1) At a fine selection scale, the red duikers species reduce competition by temporal partitioning (*C. castaneus*), and by the selection of specific food resources (*C. callipygus*, and *C. leucogaster*); (2) at an intermediate selection scale, the co-existence of *P. congica*, red duikers and *C. silvicultor* could be linked to the selection of distinct feeding sites imposed by fruit size; (3) and on a large spatial selection scale, *C. nigrifrons* selects a specific habitat (swampy areas).

Finally, results of this study highlight the potential of camera traps for modelling activity patterns and interactions between sympatric species. In addition, our results provide important information for better conservation and management strategies of duiker populations. For instance, regulation of hunting activities should better integrate activity patterns of each species, which is not currently the case.

## 5. Conclusions

Data from 315 camera traps allowed the quantification of activity patterns of a community of duiker species. We studied the extent of overlapping activity patterns and co-occurrence for duikers for the first time in Central Africa. More specifically, we gathered important information on two poorly-known species, namely *C. leucogaster* and *C. nigrifrons*. A clarification was also made on the activity patterns of *C. silvicultor* which was previously subject to controversy.

In view of these results, we consider camera traps to be suitable tools for studying the behaviour, in particular the activity patterns of duikers, which are elusive mammals difficult to observe in their natural environment. Camera traps do not, however, make it possible to determine the influence of weight, age and sex for these species, as such observations are difficult to detect on the images.

In terms of prospects, in-depth studies on the variation of activity patterns according to anthropogenic activities, climate, habitat, predators, and the existence of possible commensal relationships with other taxa seem particularly appropriate.

## Figures and Tables

**Table 1:** Number of independent detection events per site and species.

Sites	*Cephalophus callipygus ^R^*	*C. castaneus ^R^*	*C. leucogaster ^R^*	*C. nigrifrons ^R^*	*C. silvicultor ^Y^*	*Philantomba congica ^B^*
Dja CMR	70	30	0	1	60	417
Makalaya CMR	140	30	1	1	32	151
Mindourou 1 CMR	7	13	0	0	6	42
Mindourou 2 CMR	27	17	0	1	14	99
Mindourou 3 CMR	12	13	0	0	4	86
Ingolo COG	1	35	0	0	10	113
Ngombe COG	230	148	1	0	55	545
Bambidie GBN	100	45	12	1	14	52
Djoutou GBN	102	31	19	3	19	359
Ndambie GBN	116	67	25	0	30	77
Ovan GBN	140	36	3	8	35	601
Maduda RDC	0	4	0	27	0	20
Total	945	469	61	42	279	2562

CMR: Cameroun, GBN: Gabon, COG: Congo, DRC: Democratic Republic of Congo, R: Red duiker, Y: Yellow-backed duiker, B: Blue duiker.

**Table 2:** Estimates of species co-occurrence patterns between the species pairs. The upper half table in light grey presents the values of probabilities *p_lt_* and *p_gt_*. The lower half table in dark grey indicates standardised effect sizes of co-occurrence (positive and negative values indicated positive and negative associations respectively). Statistically significant values of effect sizes and probabilities (*p* < 0.05) are in bold type.

	*C. callipygus*	*C. castaneus*	*C. leucogaster*	*C. silvicultor*	*P. congica*
***C. callipygus***		*p_lt_* = 0.999 ***p_gt_* < 0.001**	*p_lt_* = 0.991 ***p_gt_* = 0.025**	*p_lt_* = 0.999 ***p_gt_* < 0.001**	*p_lt_* = 0.999 **p*_gt_* = 0.001**
***C. castaneus***	**0.048**		*p_lt_* = 0.875 *p_gt_* = 0.229	*p_lt_* = 0.962 *p_gt_* = 0.062	*p_lt_* = 0.875 *p_gt_* = 0.199
***C. leucogaster***	**0.017**	0.007		*p_lt_* = 0.105 *p_gt_* = 0.956	*p_lt_* = 0.958 *p_gt_* = 0.141
***C. silvicultor***	**0.056**	0.022	−0.011		*p_lt_* = 0.735 *p_gt_* = 0.376
***P. congica***	**0.034**	0.010	0.008	0.005	

## Supplementary Materials

The following are available online at https://www.mdpi.com/2076-2615/10/12/2200/s1, Table S1: Scientific names and IUCN red list categories of the studied duikers. In the absence of consensus this paper is based on the taxonomy proposed by Groves and Grubb (2011); Table S2: Sunrise and sunset variation for each site according to sampling periods; Figure S1: Activity patterns of six duikers of the Central African rainforests and activity levels. The curves are fitted circular kernel distributions, the steps are observed frequencies. Estimates of activity level, Standard Error (SE), 95% Confidence Intervals (CI) derived from the fitted distributions are bold. See Table 1 for sample sizes; Figure S2: Empirical Cumulative Distribution Function (ECDF) of *C. crusalbum* (*n* = 93, total events observed), and *C. callipygus* (*n* = 75, total events where *C. crusalbum* was present). Watson’s two-sample test *U^2^* = 0.04, *p* > 0.1.
