# Supplementary material for: Daily Activity Patterns and Co-Occurrence of Duikers Revealed by an Intensive Camera Trap Survey across Central African Rainforests
Source: Animals (Basel). 2020 Nov 24;10(12):2200. doi: 10.3390/ani10122200 (PMC7759945; doi:10.3390/ani10122200)
Supplement: Supplementary file 1 [file animals-10-02200-s001.pdf]

animals

MDPI

Supplementary Materials: Daily Activity Patterns and Co-Occurrence of Duikers Revealed by an Intensive Camera Trap Survey across Central African Rainforests

Table S1. Scientific names and IUCN red list categories of the duikers studied. In the absence of consensus this paper is based on the taxonomy proposed by Groves & Grubb (2011).

Scientific Name (Groves & Grubb, 2011)	Scientific Name (IUCN)	Groups (Colors and sizes)	Red List Category (IUCN)
Cephalophus callipygus	*Cephalophus callipygus*	red duiker (medium duiker up to 25kg)	Least Concern
Cephalophus castaneus	*Cephalophus dorsalis*	red duiker (medium duiker up to 25kg)	Near Threatened
Cephalophus crusalbum	*Cephalophus ogilbyi*	red duiker (medium duiker up to 25kg)	Least Concern
Cephalophus leucogaster	*Cephalophus leucogaster*	red duiker (medium duiker up to 25kg)	Near Threatened
Cephalophus nigrifrons	*Cephalophus nigrifrons*	red duiker (medium duiker up to 25kg)	Least Concern
Cephalophus silvicultor	*Cephalophus silvicultor*	yellow-backed duiker (large duiker: 80kg)	Near Threatened
Philantomba congica	*Philantomba monticola*	blue duiker (small duiker up to 10kg)	Least Concern

Groves, C.; Grubb, P. *Ungulate Taxonomy*. Johns Hopkins University Press: Baltimore, Maryland, 2011, p.317.

Table S2. Sunrise and sunset variation for each site according to sampling periods.

Sites	Sampling periods	Min _Sunrise	Max _Sunrise	Min _Sunset	Max _Sunset
Dja CMR	April-July 2017	05:57	06:08	18:13	18:23
Makalaya (A) CMR	Apr-June 2016	05:51	06:02	18:07	18:16
Makalaya (B) CMR	Feb-June 2018	05:51	06:17	18:07	18:17
Mindourou 1 CMR	Feb-May 2017	05:54	06:20	18:10	18:20
Mindourou 2 (A) CMR	May-July 2017	05:53	06:04	18:10	18:21
Mindourou 2 (B) CMR	Jan-Apr 2018	05:56	06:20	18:07	18:20
Mindourou 3 CMR	Mar-June 2017	05:53	06:16	18:10	18:19
Ingolo COG	May-Aug 2014	06:03	06:13	18:01	18:11
Ngombe COG	May-Aug 2014	05:49	05:59	17:58	18:09
Bambidie GBN	Nov2018-Fev 2019	05:47	06:17	17:52	18:22
Djoutou GBN	April-Aug 2014	06:02	06:12	18:04	18:14
Ndambie GBN	Oct2018-Janv 2019	05:48	06:19	17:56	18:24
Ovan GBN	May-Sept 2014	05:58	06:14	18:04	18:21
Maduda RDC	April-July 2014	06:07	06:18	18:00	18:13

CMR: Cameroun, GBN: Gabon, COG: Congo, DRC: Democratic Republic of Congo.

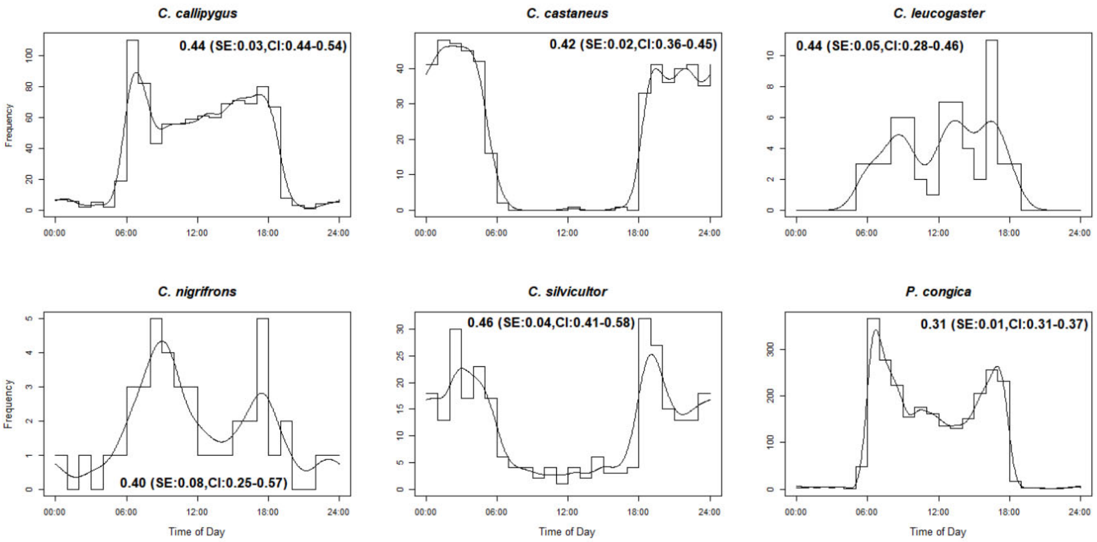

Figure S1. Activity patterns of six duikers of the Central African rainforests and activity levels. The curves are fitted circular kernel distributions, the steps are observed frequencies. Estimates of activity level, Standard Error (SE), 95% Confidence Intervals (CI) derived from the fitted distributions are bold. See table 1 for sample sizes.

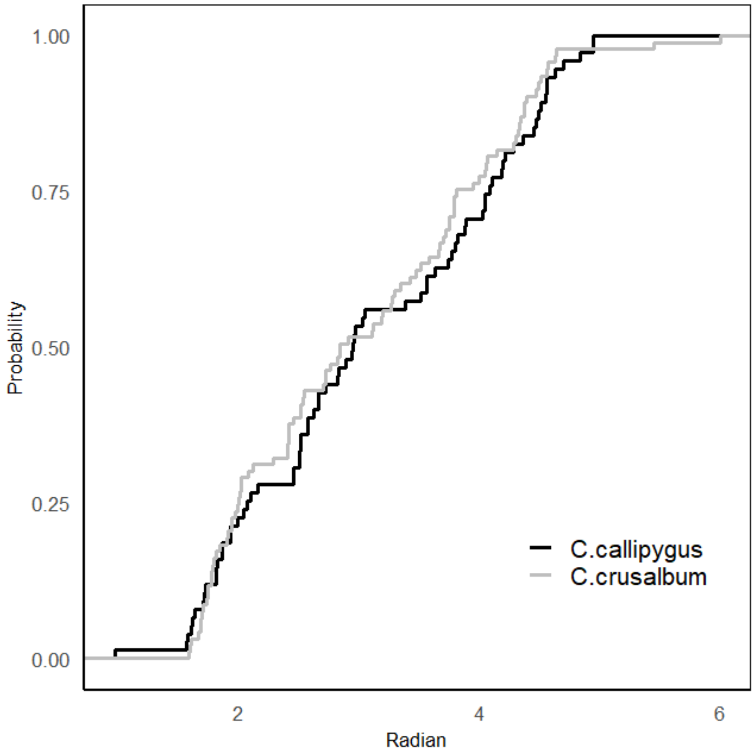

Figure S2. Empirical Cumulative Distribution Function (ECDF) of *C. crusalbum* (n = 93, total events observed), and *C. callipygus* (n = 75, total events where *C. crusalbum* was present). Watson's two-sample test $U^2 = 0.04$, $p > 0.1$.
